# Temperature-Controlled Immobilization for Testicular Irradiation: A Technical Report of a Single Case

**DOI:** 10.3390/jcm15103568

**Published:** 2026-05-07

**Authors:** Byungyong Kim, Seung Gyu Park, Euncheol Choi, Sang Hee Youn, Young Rok Do, Byung Hoon Kim, Byungwook Choi, Myeongsoo Kim, Sang Jun Byun

**Affiliations:** 1 Department of Radiation Oncology, Keimyung University Dongsan Hospital, Daegu 42601, Republic of Korea; afecym@gmail.com (B.K.); psk818@dsmc.or.kr (S.G.P.); cec0510@dsmc.or.kr (E.C.); ysh@dsmc.or.kr (S.H.Y.); 2 Department of Radiation Oncology, Keimyung University School of Medicine, Daegu 42601, Republic of Korea; 3 Department of Internal Medicine, Keimyung University School of Medicine, Daegu 42601, Republic of Korea; dyr1160@dsmc.or.kr; 4 Department of Urology, Keimyung University School of Medicine, Daegu 42601, Republic of Korea; blackporori@dsmc.or.kr; 5 Department of Nuclear Medicine, Daegu Catholic University School of Medicine, Daegu 42472, Republic of Korea; nmchoibw@gmail.com

**Keywords:** primary testicular lymphoma, testicular irradiation, immobilization technique, cremasteric reflex, temperature control, cone-beam CT

## Abstract

**Background:** Testicular irradiation presents technical challenges due to the temperature-dependent cremasteric reflex causing positional variability, yet detailed immobilization protocols addressing this issue and cone-beam computed tomography (CBCT)-based setup data remain lacking. This formative and preliminary single-patient descriptive technical report describes a temperature-controlled immobilization technique and reports preliminary setup observations from its clinical application. **Methods:** A 74-year-old male with primary testicular diffuse large B-cell lymphoma (DLBCL) received prophylactic contralateral testicular irradiation. The immobilization protocol combined a custom thermoplastic device with infrared warming to maintain the scrotal surface temperature at 36–36.5 °C, intended to facilitate a relaxed scrotal position prior to and during each fraction under temperature-controlled conditions. Treatment was delivered using a three-field three-dimensional conformal radiotherapy (3D-CRT) technique (30.6 Gy in 17 fractions), and seven CBCT scans were used to document interfraction setup measurements. **Results:** The treatment was completed as planned with adequate target coverage (clinical target volume [CTV] D97% = 100%) and minimal organ-at-risk (OAR) doses. Setup measurements showed a CTV root-mean-square displacement (RMS) of 3.8 mm and a mean Dice similarity coefficient (DSC) of 0.85, while the testis alone showed an RMS of 5.2 mm and a mean DSC of 0.73. **Conclusions:** The temperature-controlled immobilization technique was feasibly implemented, and the setup measurements observed during its application showed a CTV RMS of 3.8 mm and a mean DSC of 0.85. These findings may provide a practical reference for institutions encountering this rare clinical scenario.

## 1. Introduction

Primary testicular lymphoma (PTL) is a rare extranodal manifestation of non-Hodgkin lymphoma, accounting for approximately 1–2% of all non-Hodgkin lymphomas, and predominantly occurs in elderly men aged 60–70 years [1,2,3,4]. Histologically, diffuse large B-cell lymphoma (DLBCL) comprises approximately 80–90% of cases, and PTL exhibits an aggressive clinical course characterized by a high frequency of systemic dissemination, extranodal involvement, and poor prognosis [5,6,7].

PTL demonstrates a propensity for relapse in immune-privileged sanctuary sites, particularly the contralateral testis and central nervous system (CNS). The blood–testis barrier limits the penetration of systemic chemotherapy agents to therapeutic concentrations, and the cumulative risk of contralateral testicular relapse reaches approximately 42% at 15 years without prophylactic radiotherapy [7,8]. A phase II trial conducted by the International Extranodal Lymphoma Study Group (IELSG) demonstrated that combined treatment with rituximab-CHOP, intrathecal methotrexate, and contralateral testicular irradiation (25–30 Gy) achieved five-year progression-free and overall survival rates of 74% and 85%, respectively [9]. Based on evidence from prospective trials, prophylactic testicular radiotherapy of 25–30 Gy has been recommended for PTL patients regardless of disease stage [9,10].

However, testicular irradiation presents inherent technical challenges. The scrotal position is intrinsically unstable, with significant variations in shape and position primarily mediated by the temperature-dependent reflex of the cremaster muscle [11,12]. The physiological function of this reflex is to maintain an optimal temperature of approximately 34 °C for spermatogenesis, elevating the testes toward the body in cold environments and lowering them in warm conditions [12,13]. Although the cremasteric reflex can also be elicited by tactile stimulation, emotional stress, and anxiety [12], local thermoreceptors in the scrotal skin are considered the dominant afferent input regulating the tone of the dartos and cremaster muscles [14], and tissue-level studies have further suggested that local temperature directly modulates cremasteric contractile properties [15]. This involuntary movement poses a substantial obstacle to maintaining consistent patient positioning across treatment fractions.

Previous literature has reported various approaches to scrotal irradiation. Brouwer et al. conducted an *in silico* planning study comparing beam arrangements and reported that an oblique photon beam configuration with two wedge filters achieved superior planning target volume (PTV) coverage and minimal maximum dose; however, setup reproducibility data in actual patients were not included [11]. Patro et al. introduced the “Burger Technique,” which combines a thermoplastic mask with a wax bolus, but the report was limited to a brief procedural overview without detailed protocol parameters for clinical reproduction [16]. The International Lymphoma Radiation Oncology Group (ILROG) has emphasized the importance of computed tomography (CT)-based treatment planning and appropriate patient immobilization for testicular irradiation, yet specific guidance on scrotal immobilization methods remains limited [17,18]. Although these contributions have advanced the field, a common limitation is the lack of detailed, reproducible immobilization protocols that address the cremasteric reflex and can be readily adopted by other institutions.

Cone-beam CT (CBCT) has become an essential tool for image-guided radiotherapy (IGRT), enabling visualization of interfraction positional changes [19,20]. In pelvic radiotherapy, CBCT-based studies have reported systematic errors of 2–3 mm and random errors of 3–4 mm as general reference values [21]. For testicular irradiation, however, no CBCT-based setup observations have been reported to date, partly reflecting the rarity of this clinical scenario.

In this preliminary technical report, we describe a temperature-controlled immobilization protocol designed to minimize potential scrotal mobility caused by the cremasteric reflex during testicular irradiation. The protocol employs infrared warming to maintain the scrotal surface temperature at 36–36.5 °C, combined with a custom thermoplastic device, in a three-field three-dimensional conformal radiotherapy (3D-CRT) setting. The quantified interfraction setup variability observed while employing this protocol is reported, and this single-patient descriptive study serves as a formative reference for institutions encountering this rare clinical scenario.

## 2. Materials and Methods

### 2.1. Patient and Clinical Context

A 74-year-old man presented to a local urology clinic with a one-month history of right scrotal swelling. Scrotal ultrasonography revealed a heterogeneous 3 cm mass in the right testis with associated fluid collection, raising suspicion for testicular cancer. The patient was referred to our institution’s Department of Urology for further evaluation and management.

His past medical history was notable for a transurethral resection of the prostate (TURP) performed 14 years earlier for benign prostatic hyperplasia, and he had been on medication for diabetes mellitus. On physical examination, a 5 cm right testicular mass was palpated. Serum tumor markers were within normal limits. The patient subsequently underwent a right orchiectomy, and histopathological examination confirmed a diagnosis of diffuse large B-cell lymphoma (DLBCL).

For additional evaluation and management, he was referred to the Department of Hemato-Oncology. Two weeks after the orchiectomy, staging studies including neck, chest, abdominal, and pelvic CT scans, as well as positron emission tomography–CT (PET-CT), revealed no evidence of residual or metastatic disease. Accordingly, the patient was classified as having Ann Arbor stage I(E) primary testicular lymphoma. Serial imaging performed after surgery confirmed sustained no evidence of disease (NED) status.

The patient subsequently received six cycles of R-CHOP chemotherapy (elderly regimen), achieving complete remission on both interim CT after three cycles and restaging PET-CT after completion of six cycles. Central nervous system (CNS) prophylaxis was not administered, considering the patient’s advanced age, concerns regarding treatment-related toxicity, and the anticipated limited tolerance to additional interventions such as intrathecal chemotherapy or high-dose methotrexate-based regimens.

Following systemic therapy, he was referred to the Department of Radiation Oncology for prophylactic irradiation of the contralateral testis. The interval between orchiectomy and radiotherapy was approximately 7–8 months, with an interval of approximately two months between completion of chemotherapy and initiation of radiotherapy.

This treatment approach—orchiectomy followed by systemic chemoimmunotherapy and consolidative radiotherapy—is consistent with current standard management strategies for primary testicular DLBCL, although omission of CNS prophylaxis represents a deviation based on individualized clinical judgment. The overall clinical course and the temporal relationship between these interventions are summarized in Table 1.

### 2.2. Patient Immobilization and Temperature Control Technique

#### 2.2.1. Patient Positioning and Immobilization Device

**Figure 1:**
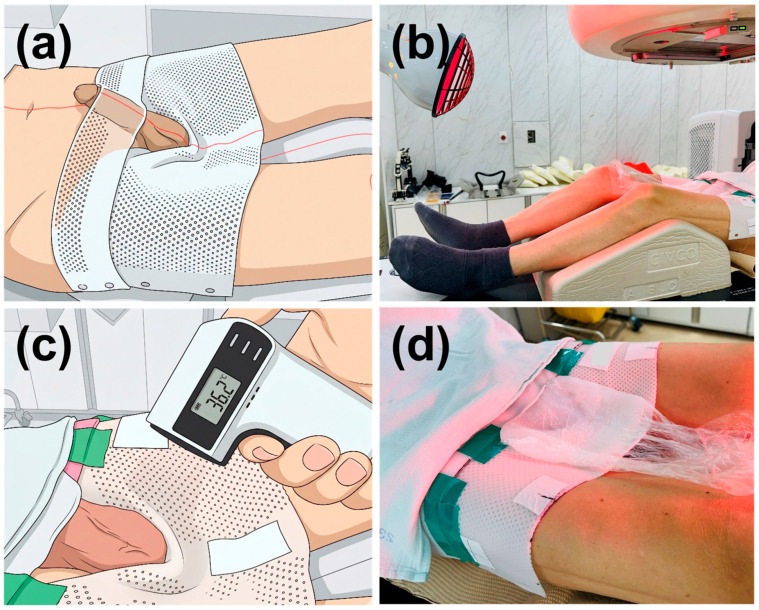
The temperature-controlled immobilization setup. (**a**) Schematic illustration of the thermoplastic mask and the patient’s setup position. (**b**) Wide-angle photograph of the treatment room, including the infrared heater placement. (**c**) Schematic representation of the surface temperature monitoring protocol using an infrared thermometer (FS-300, HuBDIC, Anyang, South Korea). (**d**) Clinical photograph showing the implementation of the protective bolus over the immobilization mask.

The patient was positioned supine with a knee support placed beneath the popliteal fossa to maintain consistent lower extremity positioning. A custom thermoplastic immobilization device was fabricated to securely immobilize the pelvis and proximal thighs bilaterally (Figure 1a). The device was molded to conform to the individual patient’s anatomical contours around the inguinal and proximal scrotal regions, providing a stable fixation base. A penile stabilization component was integrated to minimize genital movement and reduce unnecessary radiation exposure to adjacent normal tissues. The thermoplastic device was designed to leave the scrotal surface exposed to allow direct access for temperature monitoring and warming. Schematic illustrations in Figure 1a,c were generated from clinical photographs using Gemini 3.1 Pro (Google LLC, Mountain View, CA, USA) to preserve patient privacy while retaining anatomical accuracy required for protocol reproduction. All AI-generated outputs were manually reviewed by the authors.

#### 2.2.2. Temperature Control Protocol

The temperature control protocol constitutes the central component of this technique and was applied before every treatment fraction according to the following standardized procedure.

A Ministry of Food and Drug Safety (MFDS)-certified medical infrared heater (KH250, Sungrim Electronics Medical, Goyang, South Korea) was used as the warming device under continuous clinical monitoring. This device was selected for its ability to deliver uniform radiant heat without direct skin contact. The heater was positioned at the foot of the treatment table, with a distance of approximately 70 cm maintained between the infrared emission head and the scrotal surface (Figure 1b). This distance was determined empirically during the simulation session to achieve a gradual and controllable temperature rise without overheating.

Before each fraction, the scrotal surface and a 5 mm thick bolus were warmed simultaneously. The scrotal surface temperature was monitored in real-time using a non-contact infrared thermometer (FS-300, HuBDIC, Anyang, South Korea). Approximately 2–3 min of warming were required to reach the target temperature of 36–36.5 °C (Figure 1c). This target range (36–36.5 °C) was chosen to approximate core body temperature. Physiologically, when the scrotal surface temperature reaches this threshold, the body’s heat-dissipation response is triggered, inducing maximal relaxation of the cremaster muscle to move the testes away from the body [12,13,22]. By targeting the dominant thermal sensors in the skin, this non-invasive surface warming is intended to modulate the reflex arc regardless of the temperature of the testicular parenchyma or deep blood flow. Once the target temperature was confirmed, the pre-warmed bolus was applied to the scrotal surface and radiation delivery was initiated (Figure 1d). The infrared heater was kept in operation continuously throughout beam delivery to maintain the scrotal surface temperature at the target range.

#### 2.2.3. Rationale for Protocol Design and Modifications from Prior Techniques

This technique was developed as a modification of the previously reported “Burger Technique” by Patro et al., which combines a thermoplastic mask with a wax bolus for scrotal immobilization [16]. The present protocol differs in three key aspects. First, the thermoplastic coverage over the scrotal region was intentionally removed to enable real-time temperature monitoring and direct visual confirmation of scrotal position during warming. Second, infrared warming was introduced as an active intervention to address the cremasteric reflex, whereas the original technique relied on mechanical immobilization alone without thermal modulation. Third, limiting the immobilization device to the pelvis and thighs simplified the setup process, as scrotal thermoplastic molding in the original technique requires careful positioning that may be difficult to reproduce consistently across fractions.

The overall workflow for each treatment fraction can be summarized as follows: (1) patient positioning and thermoplastic device application, (2) infrared warming of the scrotal surface and bolus for 2–3 min with continuous temperature monitoring, (3) confirmation of target temperature (36–36.5 °C), (4) bolus application, and (5) radiation delivery with continuous infrared warming maintained throughout. This sequence was followed consistently for all 17 fractions without procedural deviation.

### 2.3. Radiation Treatment Planning

Considering the potential for microscopic disease spread in testicular lymphoma, the entire scrotal contents, including the remaining left testis (epididymis and spermatic cord), were designated as the clinical target volume (CTV). Due to the anatomical constraints of the scrotum, a significant portion of a potential PTV would extend beyond the body contour into the air; therefore, a separate PTV was not defined. Instead, to account for setup uncertainties and involuntary movements, including the cremasteric reflex, generous field margins were empirically applied directly to the CTV. These margins were set at 1.5 cm laterally and 2.0 cm in the superior-inferior direction to provide a robust safety buffer. Organs at risk (OARs), including the rectum, bladder, bilateral femoral heads, and seminal vesicles, were contoured.

**Figure 2:**
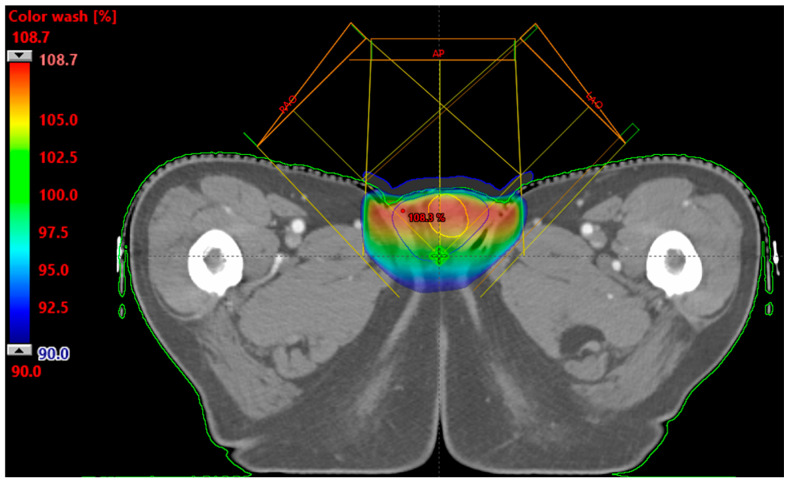
Three-dimensional conformal radiotherapy plan. Testicular irradiation plan using a three-field technique with one anteroposterior (AP) field and two oblique fields (LAO/RAO, 45°/315°) delivering 30.6 Gy in 17 fractions.

Treatment planning was performed using the Eclipse treatment planning system (version 15.5, Varian Medical Systems, Palo Alto, CA, USA) with a 3D-CRT technique using 6 MV photons on a Clinac iX linear accelerator equipped with a Millennium MLC (Varian Medical Systems, Palo Alto, CA, USA). A three-field arrangement consisting of one anteroposterior (AP) field and two oblique fields (left anterior oblique 45°, right anterior oblique 315°) was utilized (Figure 2), with wedge filters applied to all fields for dose homogeneity optimization. The oblique fields were shaped with MLC using a 2 cm margin from the CTV, while the AP field used 1.5 cm lateral and 2 cm superior-inferior margins. The prescription dose was 30.6 Gy delivered in 17 fractions of 1.8 Gy each, based on a conventional fraction size of 1.8 Gy that results in a total dose marginally exceeding the recommended range of 25–30 Gy.

The beam arrangement was based on the findings of Brouwer et al., who reported that an oblique photon beam arrangement with two wedge filters achieved favorable PTV coverage and dose homogeneity [11]. In the present case, an additional AP field with an IN-direction wedge was included to compensate for the dose gradient along the superior-inferior axis, considering that the elongated scrotal geometry along this direction may be insufficiently covered by the two oblique fields alone. The wedge orientation was designed to deliver a higher dose toward the superior aspect, thereby counterbalancing the dose fall-off inherent to the oblique field arrangement and improving overall dose uniformity across the target volume.

### 2.4. CBCT-Based Setup Measurements

**Figure 3:**
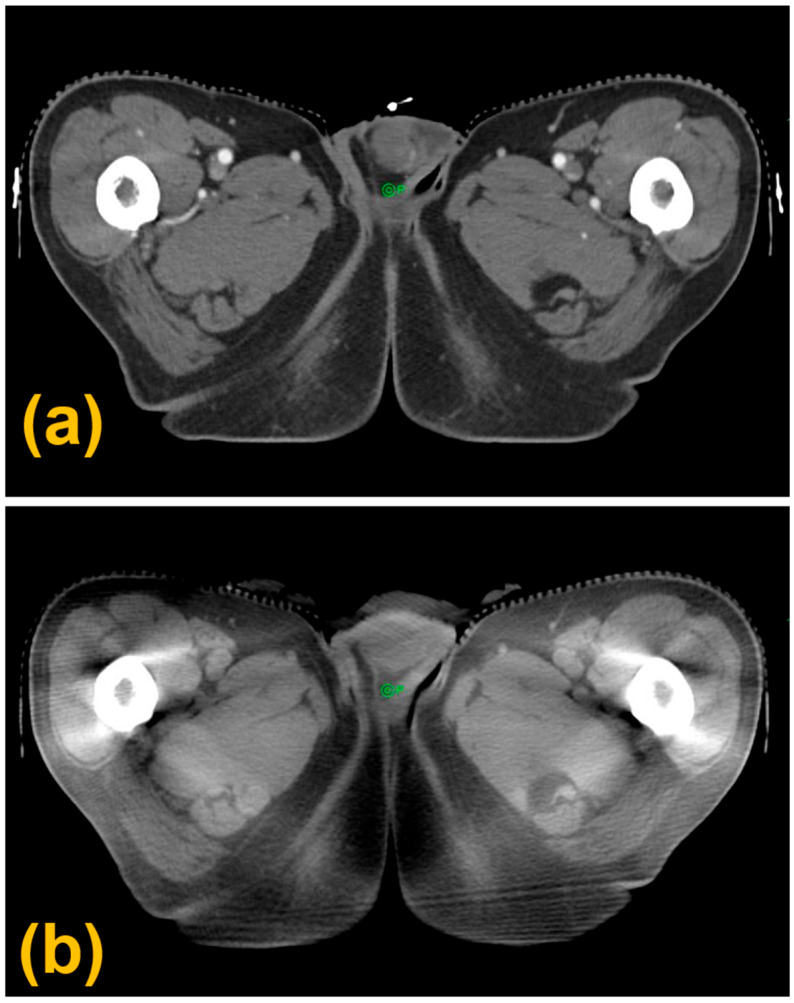
Rigid registration of planning CT (**a**) and CBCT (**b**) images. The green circle and the letter “P” denote the location of the treatment isocenter.

During the 17-fraction treatment course, CBCT scans were acquired twice weekly as part of routine image guidance, yielding a total of seven images distributed across the treatment period. These images were retrospectively reviewed to document interfraction positional changes in the target structures. A single radiation oncologist contoured the CTV and testicular structures on the planning CT and all CBCT images to ensure consistency. Rigid registration between planning CT and CBCT was performed using the Eclipse system (Varian Medical Systems, Palo Alto, CA, USA) based on the immobilization device and pelvic bony anatomy. Positional displacements of the CTV and testicular structures were then measured from the registered image sets. Figure 3 shows representative rigidly registered planning CT and CBCT images.

Interfraction positional changes were characterized using the following measurements. Three-dimensional displacements (Δ*x*, Δ*y*, Δ*z*) were obtained from the center-of-mass shift in the CTV and testis between the planning CT and each CBCT. The three-dimensional displacement magnitude (Δ*r*) and root-mean-square (RMS_3D_) were calculated as:

(1) $$∆r=\sqrt{{\left(∆x\right)}^{2}+{\left(∆y\right)}^{2}+{\left(∆z\right)}^{2}}$$

(2) $${RMS}_{3D}=\sqrt{mean({∆r}^{2})}$$

The RMS_3D_ is hereafter referred to as RMS for brevity. The Dice similarity coefficient (DSC), a standard metric of spatial overlap, was calculated using the statistics function in Eclipse to describe the geometric correspondence between structures on planning CT and CBCT:

(3) $$DSC=\frac{2\left|A\cap B\right|}{\left|A\right|+\left|B\right|}$$

Interfraction volume changes were also recorded. These metrics were selected as descriptive measures to characterize the observed setup data; no inferential statistical analysis was performed, given the single-patient design.

## 3. Results

### 3.1. Dosimetric Results

Dose-volume histogram (DVH) analysis showed that 97% of the CTV received 100% of the prescription dose (30.6 Gy), and the maximum dose within the CTV was 108.7% (33.3 Gy). All major organs at risk received minimal doses, with the rectum, bladder, femoral heads, and seminal vesicles all receiving less than 0.58 Gy. The treatment was completed as planned over 17 fractions. Retrospective clinical record review confirmed no Grade 1 or higher acute adverse events (e.g., radiation dermatitis or scrotal pain) according to the CTCAE v5.0.

### 3.2. CBCT-Based Setup Observations

The setup measurements obtained from seven CBCT scans acquired during the treatment course are summarized in Table 2.

For the CTV, the mean three-dimensional displacement was 3.6 ± 1.3 mm (maximum 5.4 mm) with an RMS of 3.8 mm. The testis showed a mean three-dimensional displacement of 4.9 ± 1.9 mm (maximum 7.4 mm) with an RMS of 5.2 mm. In both structures, the largest displacement was observed in the longitudinal (superior-inferior) direction (CTV: 3.0 ± 1.1 mm; testis: 4.4 ± 1.7 mm). Detailed directional displacement values are presented in Table 2.

Volume measurements remained stable throughout the treatment course, with variation in less than 3% for both the CTV (planning CT: 45.8 cm^3^; mean CBCT: 46.9 ± 1.1 cm^3^) and the testis (planning CT: 15.5 cm^3^; mean CBCT: 15.46 ± 0.05 cm^3^).

The mean DSC was 0.85 ± 0.04 for the CTV and 0.73 ± 0.09 for the testis. The CTV maintained a DSC above 0.80 in 6 of 7 fractions (86%), while the testis exceeded this threshold in 3 of 7 fractions (43%).

## 4. Discussion

The core concept of this technique is to promote cremaster muscle relaxation by maintaining the scrotal surface temperature at 36–36.5 °C through infrared warming. The physiological basis for this approach lies in the scrotal thermoregulatory system, in which the dartos and cremaster muscles regulate testicular position in response to temperature changes, contracting in cold conditions to elevate the testes and relaxing in warm conditions to allow testicular descent [12,22]. Although the cremasteric reflex is known to respond to multiple afferent inputs, a hierarchy exists among these stimuli in governing tonic scrotal position. Notably, local temperature receptors in the scrotal skin provide the dominant afferent input for regulating the tone of these muscles, with whole-body thermal signals playing a secondary role [14]. Warming the scrotal surface to near body temperature is therefore expected to promote relaxation of these muscles, which may contribute to reducing positional variability without requiring any change in core body temperature [14,22]. In the present protocol, infrared warming was maintained continuously during beam delivery, not only during the preparatory phase, with the intent of sustaining cremasteric relaxation throughout each treatment fraction.

Several practical aspects of this protocol merit discussion. As described in Section 2.2, the present protocol was developed as a modification of the “Burger Technique” by Patro et al. [16], with deliberate design changes to enable real-time thermal monitoring, active cremasteric reflex suppression, and a simplified setup process. From an implementation perspective, the equipment used in this protocol—a commercially available medical infrared heater and a non-contact infrared thermometer—is commonly available in most radiotherapy departments. The additional time required for the warming procedure was approximately 2–3 min per fraction, which represents a modest addition to the standard setup workflow. No warming-related adverse events were documented on retrospective clinical record review, and no significant patient discomfort was reported during routine pre- and post-fraction assessments by the radiation oncologist and therapists, although a formal patient-reported outcome measure such as the Numeric Rating Scale (NRS) was not employed. The absence of documented acute toxicities is likely attributed to the limited target volume and the non-contact nature of the warming technique. These factors suggest that the described technique may be adopted without high additional cost or workflow disruption, which is a relevant consideration given that institutions may encounter this rare clinical scenario infrequently and may not have dedicated scrotal immobilization equipment.

Approaches to scrotal stabilization can be categorized into mechanical and physiological methods. Recently, Yamaguchi et al. reported a mechanical approach using a custom-made immobilizer that physically pulls the scrotum cranially to minimize involuntary motion for high-resolution MRI [23]. While effective for local motion suppression, such a physical constraint may pose challenges in the radiotherapy setup. In contrast, the physiological approach of using warmth is supported by the European Society of Urogenital Radiology (ESUR) guidelines, which recommend placing warm towels over the scrotum to reduce muscular contractions [24]. Our protocol builds upon this established principle by utilizing active infrared warming, offering a non-contact and highly reproducible alternative that is intended to promote maximal cremasteric relaxation without mechanical interference.

In our CBCT-based setup observations, both the CTV and the testis exhibited the largest displacement in the longitudinal direction. Physiologically, the scrotum is a highly mobile structure capable of shifting by several centimeters (up to 3–5 cm in adults) in response to thermal stimuli [11,25,26]. Notably, under this protocol, the CTV longitudinal displacement was observed to be 3.0 ± 1.1 mm on average, a magnitude smaller than the physiological mobility range described above. While a causal contribution of active thermal modulation cannot be inferred from a single case, this observation is compatible with the hypothesis that thermal modulation may attenuate involuntary motion along this primary vector. This directional predominance is consistent with the primary vector of cremasteric muscle contraction, which elevates the testis along the superior-inferior axis [12,13]. While the temperature control protocol was designed to suppress thermally mediated cremasteric activity, the cremasteric reflex is known to be elicited by multiple afferent inputs, including emotional stress, tactile stimulation, and anxiety [12], which may not be fully mitigated by thermal modulation alone. The treatment environment itself—involving positioning, exposure, and radiation delivery to a sensitive anatomical region—may have contributed to residual cremasteric activity that preferentially manifested in the longitudinal direction. The difference in setup metrics between the CTV and the testis suggests that the testis may undergo additional displacement within the scrotal sac even when the overall scrotal position is relatively maintained. From a clinical standpoint, however, when the entire scrotal contents are designated as the CTV, such internal testicular movement is inherently encompassed within the target volume. This observation may support the rationale for defining the CTV as the entire scrotum rather than the testis alone in testicular irradiation.

Previous reports on testicular irradiation have focused primarily on beam arrangement optimization or immobilization device descriptions. Brouwer et al. conducted an *in silico* planning comparison without clinical setup data [11], and Patro et al. described the “Burger Technique” as a procedural overview without reporting interfraction positional measurements [16]. The present report adds to these contributions by providing a detailed, reproducible protocol description together with CBCT-based setup measurements from its clinical application.

While direct comparison of the observed values with existing literature is limited by differences in anatomical site, measurement methodology, and patient population, placing them in a broader context may be informative. In pelvic radiotherapy, setup errors are known to vary with immobilization method and treatment site [27], and Ye et al. reported a mean 3D displacement of 3.5 mm using fiducial markers in prostate cancer patients [28]. While no definitive causal relationship can be established due to the single-case design, the quantified setup stability observed under this protocol, with a CTV RMS of 3.8 mm, provides a preliminary frame of reference for this rare clinical scenario. This value falls within the 2–4 mm range of setup errors typically reported for rigid pelvic structures in IGRT studies. Observing setup measurements within this range in the highly mobile scrotal region, where movement potential is physiologically considerably greater, may indicate the technical feasibility of the temperature-controlled approach in this single case, although direct equivalence between these clinical scenarios cannot be claimed and broader efficacy cannot be inferred without comparative data. The present study employed a 3D conformal technique, which generally accommodates larger positional uncertainties than intensity-modulated approaches. Should highly conformal techniques be considered for testicular irradiation in the future, more precise positional data from multiple patients would be needed.

Several limitations should be acknowledged. The findings are derived from a single patient, and the contribution of temperature control to the observed positional stability could not be isolated from other factors, including the thermoplastic immobilization device, patient positioning, and individual anatomical characteristics. Comparative data obtained with and without infrared warming would be necessary to establish the independent effect of thermal modulation, which was beyond the scope of this report. Intrafraction motion was not assessed; although the infrared heater was maintained during beam delivery, formal temperature and position monitoring during irradiation were not performed, and the degree to which cremasteric relaxation was sustained throughout each fraction remains unknown. Additionally, the CBCT-based measurements were obtained from seven of 17 fractions, and the representativeness of this sample for the entire treatment course cannot be confirmed. The contouring of scrotal structures on CBCT images is subject to inherent uncertainty due to the limited soft-tissue contrast of CBCT, which may have influenced the DSC and displacement values.

These limitations are consistent with the intent of this formative and preliminary report, which is to provide a single-patient descriptive account of a feasible immobilization protocol in sufficient detail for clinical reproduction and to document the setup measurements observed during its application. Given the extreme rarity of primary testicular lymphoma, this preliminary technical description may contribute to building collective knowledge. In this context, detailed single-case technical reports that provide specific protocol parameters and observational data may serve a practical role by offering a starting point for institutions encountering this clinical scenario and by contributing to the gradual accumulation of shared clinical experience across centers.

## 5. Conclusions

This technical note describes a temperature-controlled immobilization technique for prophylactic contralateral testicular irradiation, in which infrared warming was used to maintain the scrotal surface temperature at 36–36.5 °C with the intent of attenuating the cremasteric reflex. The protocol was feasibly implemented using readily available equipment with minimal additional setup time, and the treatment was completed over 17 fractions without procedural deviation. Retrospective clinical record review identified no Grade 1 or higher acute adverse events according to the CTCAE v5.0. The quantified setup stability observed under this protocol, with a CTV RMS of 3.8 mm and a mean DSC of 0.85, suggests that the technique is feasible. The greater positional variability of the testis alone may support defining the entire scrotum as the CTV. The specific protocol parameters described here—including the warming procedure, target temperature range, equipment setup, and continuous infrared heating during beam delivery—are intended to provide a practical reference for institutions encountering this rare clinical scenario and to contribute to the gradual accumulation of shared experience toward future protocol refinement in testicular irradiation.

## Figures and Tables

**Table 1:** Timeline of the clinical course and treatment interventions.

Time from Orchiectomy	Clinical Event
Initial	Right orchiectomy performed; pathology confirmed DLBCL
Within 2 weeks	Staging CT and PET-CT: no residual disease (Ann Arbor stage I(E))
Months 1–5	Six cycles of R-CHOP chemotherapy (elderly regimen)
Post-chemotherapy	Complete remission confirmed on imaging (CT and PET-CT)
Months 7–8	Prophylactic irradiation of the contralateral testis
Follow-up	No evidence of disease (NED) status on post-treatment imaging; no significant treatment-related toxicity observed

**Table 2:** Interfraction setup measurements and geometric indices obtained from serial CBCT imaging.

Parameter	CTV	Testis
**Positional displacement (mm)**		
Lateral (*x*)	0.6 ± 1.2 (range: −1.2 to 2.6)	0.3 ± 1.5 (range: −2.2 to 2.2)
Vertical (*y*)	−1.2 ± 1.0 (range: −2.5 to 0.6)	0.7 ± 1.7 (range: −0.8 to 4.3)
Longitudinal (*z*)	3.0 ± 1.1 (range: 1.5 to 4.6)	4.4 ± 1.7 (range: 2.0 to 7.0)
3D displacement (Δ*r*)	3.6 ± 1.3 (range: 1.7 to 5.4)	4.9 ± 1.9 (range: 2.3 to 7.4)
Maximum 3D displacement	5.4	7.4
RMS (mm)	3.8	5.2
**Volume measurements**		
Planning CT volume (cm^3^)	45.8	15.5
Mean CBCT volume (cm^3^)	46.9 ± 1.1	15.46 ± 0.05
Volume variation (%)	2.4 ± 2.4	−0.3 ± 0.3
Range of volume (cm^3^)	45.6–48.1	15.4–15.5
**Geometric conformity**		
Dice similarity coefficient	0.85 ± 0.04 (range: 0.79–0.90)	0.73 ± 0.09 (range: 0.61–0.84)
Fractions with DSC > 0.80	6/7 (86%)	3/7 (43%)

## Data Availability

The data presented in this study are available on request from the corresponding author. The data are not publicly available due to patient privacy restrictions.
